# Cellular Response to Titanium Dioxide Nanoparticles in Intestinal Epithelial Caco-2 Cells is Dependent on Endocytosis-Associated Structures and Mediated by EGFR

**DOI:** 10.3390/nano7040079

**Published:** 2017-04-07

**Authors:** Kristin Krüger, Katrin Schrader, Martin Klempt

**Affiliations:** Max Rubner-Institut (MRI), Federal Research Institute for Nutrition and Food, Department of Safety and Quality of Milk and Fish Products, Hermann-Weigmann-Straße 1, 24103 Kiel, Germany; kruegerkristin@gmx.net (K.K.); katrin.schrader@mri.bund.de (K.S.)

**Keywords:** titanium dioxide nanoparticles, intestinal epithelial cells, inflammation, endocytosis, EGFR, ERK1/2

## Abstract

Titanium dioxide (TiO_2_) is one of the most applied nanomaterials and widely used in food and non-food industries as an additive or coating material (E171). It has been shown that E171 contains up to 37% particles which are smaller than 100 nm and that TiO_2_ nanoparticles (NPs) induce cytotoxicity and inflammation. Using a nuclear factor Kappa-light-chain enhancer of activated B cells (NF-κB) reporter cell line (Caco-2^nfkb-RE^), Real time polymerase chain reaction (PCR), and inhibition of dynamin and clathrin, it was shown that cellular responses induced by 5 nm and 10 nm TiO_2_ NPs (nominal size) depends on endocytic processes. As endocytosis is often dependent on the epithelial growth factor receptor (EGFR), further investigations focused on the involvement of EGFR in the uptake of TiO_2_ NPs: (1) inhibition of EGFR reduced inflammatory markers of the cell (i.e., nuclear factor (NF)-κB activity, mRNA of IL8, CCL20, and CXCL10); and (2) exposure of Caco-2 cells to TiO_2_ NPs activated the intracellular EGFR cascade beginning with EGFR-mediated extracellular signal-regulated kinases (ERK)1/2, and including transcription factor ELK1. This was followed by the expression of ERK1/2 target genes CCL2 and CXCL3. We concluded that TiO_2_ NPs enter the cell via EGFR-associated endocytosis, followed by activation of the EGFR/ERK/ELK signaling pathway, which finally induces NF-κB. No changes in inflammatory response are observed in Caco-2 cells exposed to 32 nm and 490 nm TiO_2_ particles.

## 1. Introduction

Titanium dioxide (TiO_2_) is one of the most applied nanomaterials and is widely used in the food and non-food industry as additive or coating material (for review see [1]). Food-grade TiO_2_ is coded E171 and contains up to 37% nanoparticles (NP), resulting in an estimated exposure to TiO_2_ NPs of 1 mg kg^−1^·day^−1^ in adults and up to 2–3 mg kg^−1^·day^−1^ in children [2].

Studies using Caco-2 cell lines have shown that TiO_2_ NPs induced oxidative damage [3], influenced metabolic activity and cytotoxicity [4,5], produced reactive oxygen species (ROS) [6,7], and induced the expression of interleukin 8 (IL8) [8] through the activation of nuclear factor (NF)-κB and p38 mitogen activated protein kinase (MAPK) pathways [9]. Any influence of TiO_2_ NPs on Caco-2 cells is caused by primary contact, which is followed by an interaction which is still unclear. Several studies discuss a possible activation of toll-like receptors (TLR) [10,11] or suggest an endocytic uptake [12,13,14,15]. In vitro studies on Caco-2 cells demonstrated that TiO_2_ NPs are taken up by the cell: TiO_2_ NPs have been detected intracellularly [16], surrounded by cytoplasmic vesicles [17] without disruption of junctional complexes [18]. Endocytosis, as the initial cellular reaction and beginning of the inflammatory response of TiO_2_ NPs, has not been investigated so far.

**Figure 1:**
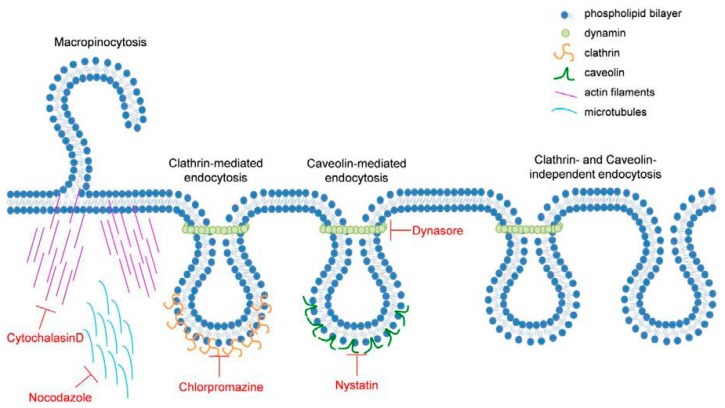
Illustration of different manners in which endocytosis occurs. Shown are the different methods of endocytosis and the mechanisms of action for the inhibitors used in this study. The illustration has been adapted from [19].

Several mechanisms responsible for cellular uptake of materials are named endocytosis: phagocytosis, which is reserved by specialized cell types and pinocytosis, which can be divided in micropinocytosis, clathrin-mediated endocytosis, caveolae-mediated endocytosis, and endocytosis independently of clathrin and caveolae [19]. Figure 1 provides an overview of the different endocytosis pathways and the inhibitors used in this study.

In the present study, we exposed Caco-2 cells to TiO_2_ particles with nominal particle diameters of 5 nm, 10 nm, 32 nm, and 490 nm. We could show that TiO_2_ NPs of 5 nm and 10 nm express their inflammatory potential mainly via an EGFR-mediated endocytosis process. Actin filaments and tubulin microtubules, as well as dynamin and, partly, clathrin, are involved in this process. Further investigations revealed that TiO_2_ NPs induced an EGFR dependent activation of downstream ERK1/2 and of the ERK-related transcription factor ELK1 (ETS (E26 transformation-specific) domain-containing protein) and consequently an increased expression of chemokine (C–C motif) ligand 2 (CCL2) and chemokine (C–X–C motif) ligand 3 (CXCL3) genes.

## 2. Results

### 2.1. Characterization of TiO_2_ Particles in Cell Medium

**Figure 2:**
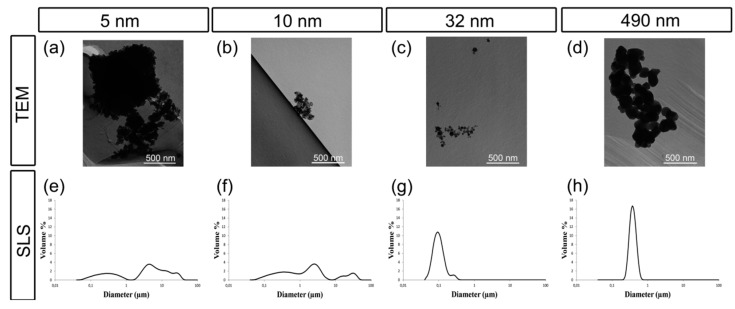
Characterization of TiO_2_ particles used in this study. (**a**–**d**) TEM images of TiO_2_ particles in medium of nominal (**a**) 5 nm (NP1); (**b**) 10 nm (NP2); (**c**) 32 nm (NP3); and (**d**) 490 nm (NP4) size. The bar corresponds to 500 nm. (**e**–**h**) Size distribution measured by static light scattering SLS of (**e**) np1; (**f**) np2; (**g**) np3; and (**h**) NP4. See text for details.

TEM of the particles in cell culture medium revealed single particle sizes of 10.7 nm (mean), 10.0 nm (median), and 9.0 nm (mode) for NP1, 12.1 nm (mean), 11.0 nm (median), and 10.0 (mode) for NP2, 23.1 nm (mean), 17.0 nm (median), 16.0 nm (mode) for NP3, and 138.9 nm (mean), 134.0 nm (median), and 78.0 nm (mode) for NP4 (Figure 2a–d). Agglomerates were observed in NP1, NP2, and NP4, while no agglomeration could be detected in NP3. By static light scattering SLS analysis (Figure 2e–h) we could demonstrate that the agglomerates of NP4 dispersed, while NP1 and NP2 showed a wide size distribution of the particles in a range from 40 nm to 57 µm with peaks at 300 nm and 4.5 µm for NP1 and peaks at 400 nm, 2.5 µm and 30 µM for NP2. NP3 and NP4 showed a unimodal size distribution with a mean agglomeration size of 96 nm for NP3 and 390 nm for NP4.

### 2.2. TiO_2_ Particles Are Located within the Cell

**Figure 3:**
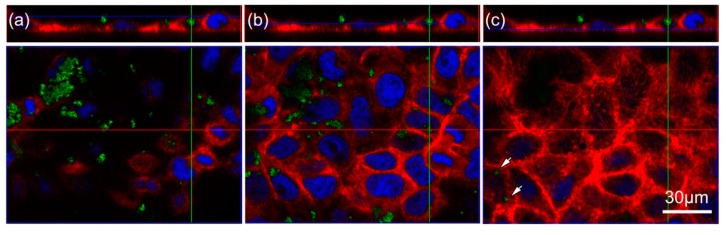
Confocal laser scanning microscopy of Caco-2 cells treated with NP2. (**a**–**c**). Monolayers of Caco-2 cells were treated with 10 nm TiO_2_ particles (NP2, concentration of 40 µg cm^−2^ of cell growth surface) and incubated for 6 h. Cells were stained for actin with Alexa Fluor 633 Phalloidin (red), nuclei were stained with DAPI (blue) and TiO_2_ NPs fluorescence (green). The upper panel shows a cross-section of Caco-2 cells. Shown are three images along the *z*-axis from the apical side (**a**) to the basal side (**c**) of the epithelium. The blue line in the upper image indicates the position of the image below, whereas the red line marks the position of the cross-section. Arrows demonstrate TiO_2_ particles within the cell membrane and intracellular. The bar corresponds to 30 µm.

Based on our previous study [9], and others [12,13,14,15], we reasoned that TiO_2_ particles are endocytosed by Caco-2 cells. To investigate this possibility, we used laser scanning confocal microscopy identification of 10 nm TiO_2_ NPs within the Caco-2 cells (Figure 3a–c). We chose 10 nm particles as these caused an inflammatory response in Caco-2 cells, as shown in our previous study [9]. Three sections along the *z*-axis ((a) apical side; (b) within; (c) basal side) of the cells provide information about TiO_2_ NPs localization. Confocal images show nuclei (blue) surrounded by actin filaments (red). Aggregates of 10 nm TiO_2_ NPs (green) are located at the apical side of the cells (Figure 3a), in the cell membrane, and within the cell (Figure 3b,c). 

### 2.3. TiO_2_ NP-Induced Inflammatory Response Is Reduced by Inhibitors of Endocytosis

**Figure 4:**
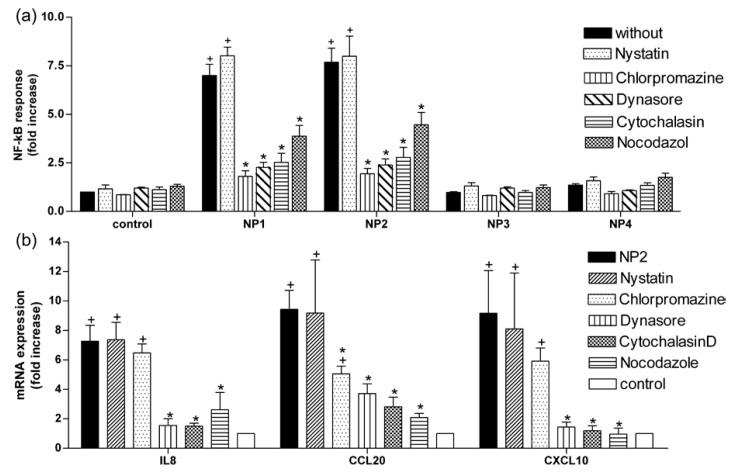
Effect of endocytic inhibitors on luciferase activity of Caco-2^nfkb-RE^ cells and expression of IL8 mRNA, CCL20 mRNA, and CXCL10 mRNA. (**a**) Treatment of nystatin (50 µg mL^−1^), chlorpromazine (10 µM), dynasore (100 µM), cytochalasin D (0.1 µg mL^−1^), and nocodazole (2 µM) on luciferase activity of Caco-2^nfkb-RE^ cells 6 h after treatment with NP1, NP2, NP3, or NP4. Before NPs treatment (concentration of 40 µg cm^−2^ of the cell growth surface) confluent monolayers of Caco-2^nfkb-RE^ cells were incubated with inhibitors. Luminescence of treated cells are presented as the fold change to untreated controls; (**b**) mRNA expression of IL8, CCL20, and CXCL10 3 h after NP2 exposure pretreated with the indicated inhibitors. mRNA expression of the treated cells are presented as the fold change to untreated controls. Mean ± SEM (*n* ≥ 3). Non-parametric analysis of variance was followed by Bonferroni post-tests (*p* < 0.05). Asterisks (*) represent significant differences to NPs treated cells, plus (+) represent significant differences to untreated controls.

To further prove that NPs are endocytosed by Caco-2 cells, we used inhibitors of molecules which are involved in endocytosis (see Figure 1) and measured the NF-κB response of Caco-2 cells after administration of TiO_2_ NPs. NP1 and NP2 induced NF-κB activity in Caco-2^nfkb-RE^ cells by a factor of 7.0 ± 0.6 and 7.7 ± 0.7, respectively, NP3 and NP4 showed no effect on NF-κB activity (1.0 ± 0.0 and 1.4 ± 0.1, respectively) (Figure 4a). Nystatin (50 µg mL^−1^), a caveolae-mediated endocytosis inhibitor, had no effect on NP-induced NF-κB activation. Treatment with 10 µM chlorpromazine (a clathrin-mediated endocytosis inhibitor) reduced NP-induced luciferase activity by 74% and 75% in NP1 and NP2, respectively. Dynasore (100 µM) (a dynamin inhibitor) reduced activation of NF-κB by 68% and 69% in NP1 and NP2, respectively. As actin filaments and microtubules play an essential role in all types of endocytosis, we investigated the effects of cytochalasin D (0.1 µg mL^−1^) and nocodazole (2 µM) on NP-induced NF-κB response. Treatment with cytochalasin D reduced NF-κB activity by 64% in both, NP1 and NP2 treated cells. Nocodazole reduced NP-induced NF-κB activity by 45% (NP1) and 42% (NP2). Since NP2 showed pronounced effects, we chose this NP for all further experiments. Expression of IL8 mRNA, chemokine (C–C motif) ligand 20 (CCL20) mRNA and C–X–C motif chemokine 10 (CXCL10) mRNA as indicators for inflammatory response of the cell was increased by 6.7 ± 1.1-fold, 9.6 ± 1.4-fold, and 9.2 ± 2.9-fold 3 h after NP2 exposure compared to untreated control cells (Figure 4b). Co-treatment with dynasore reduced the response by 76.2%, 66.7%, and 84.3% (IL8, CCL20, CXCL10), cytochalasin D altered the expression by 77.6%, 76.3%, and 87.1% and nocodazol lowered the reduced the response cells by 59.2%, 77.3%, and 89.5%. Treatment with nystatin did not change the NP-induced increase in mRNA expression of IL8, CCL20, or CXCL20, while chlorpromazine only reduced CCL20 expression by 47.3%.

### 2.4. Activation of EGFR Is Essential for the Cellular Response to TiO_2_ NPs

**Figure 5:**
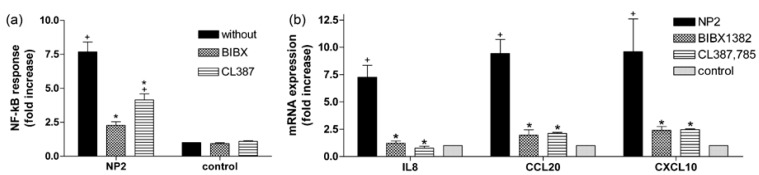
Effect of EGFR inhibitors on luciferase activity of Caco-2^nfkb-RE^ cells and expression of IL8 mRNA, CCL20 mRNA, and CXCL10 mRNA. (**a**) Effect of BIBX 1382 and CL-387785 on luciferase activity of Caco-2^nfkb-RE^ cells 6 h after NP2 (concentration of 40 µg cm^−2^ of the cell growth surface) treatment. Luminescence of treated cells are presented as the fold change to untreated control; (**b**) mRNA expression of IL8, CCL20, and CXCL10 3 h after NP2 exposure pretreated with inhibitors BIBX 1382 and CL-387785. mRNA expression of the treated cells are presented as the fold change to untreated controls. Mean ± SEM (*n* ≥ 3). Non-parametric analysis of variance was followed by Bonferroni post-tests (*p* < 0.05). Asterisks (*) represent significant differences to NP2 treated cells, plus (+) represent significant differences to untreated control cells.

As receptor mediated endocytosis is often initiated by activating the EGFR receptor, we aimed to suppress the NP-induced inflammatory response by pretreatment of Caco-2 cells with EGFR inhibitors BIBX 1382 and CL-387785. This treatment resulted in a reduced NF-κB activity in NP2 treated cells by 51.3% and 44.6%, respectively (Figure 5a), 6h after NPs exposure. Further, 3 h after NPs exposure IL8 mRNA, CCL20 mRNA, and CXCL10 mRNA expression was reduced by 81.6%, 75.3%, and 73.7%, respectively, in BIBX 1382 treated cells and by 89.1%, 77.4%, and 73.2%, respectively, in CL-387785 treated cells (Figure 5b).

### 2.5. NPs Are Colocalized with Lysosomes

**Figure 6:**
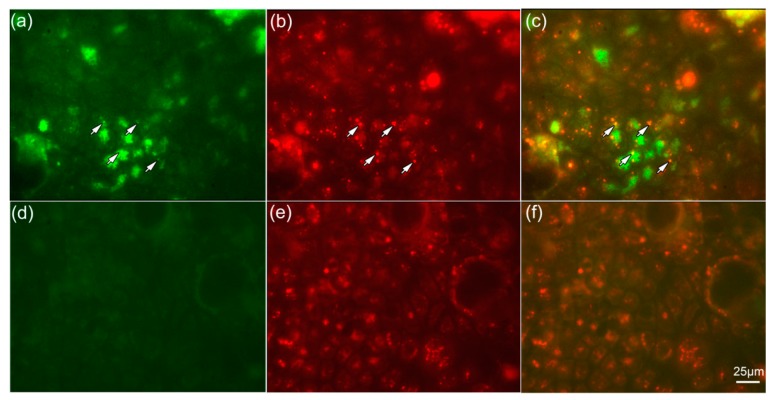
NPs are co-localized with lysosomes. Confluent monolayers of Caco-2 cells (**a**–**c**) treated with NP2 for 3 h and (**d**–**f**) untreated control cells, co-incubated with 50 nm LysoTracker^®^ Red DND-99. Fluorescence imaging of (**a**,**d**) NPs (green), visualized by using FITC filter set; (**b**,**e**) lysosomes labeled by LysoTracker^®^ Red DND-99 (red); and (**c**,**f**) overlay demonstrated co-localized NPs with lysosomes. Arrows demonstrate the same position of NPs and lysosomes. The bar corresponds to 25 µm.

To gain evidence that TiO_2_ NP2 after being taken up by endocytosis are transported though the cell within lysosomes, we used live cell imaging. Caco-2 cells treated with NP2 showed a fluorescence signal of the NPs (green, Figure 6a), whereas untreated control cells showed no signal (Figure 6d). Lysosomes labeled by LysoTracker^®^ Red DND-99 are present in NP-treated cells (Figure 6b) and in untreated control cells (Figure 6e). Overlay of both images indicate NPs localized to lysosomes 3 h after exposure (marked by the arrows, Figure 6c,f).

### 2.6. TiO_2_ NPs Activate EGFR/ERK/ELK Signaling Pathway

**Figure 7:**
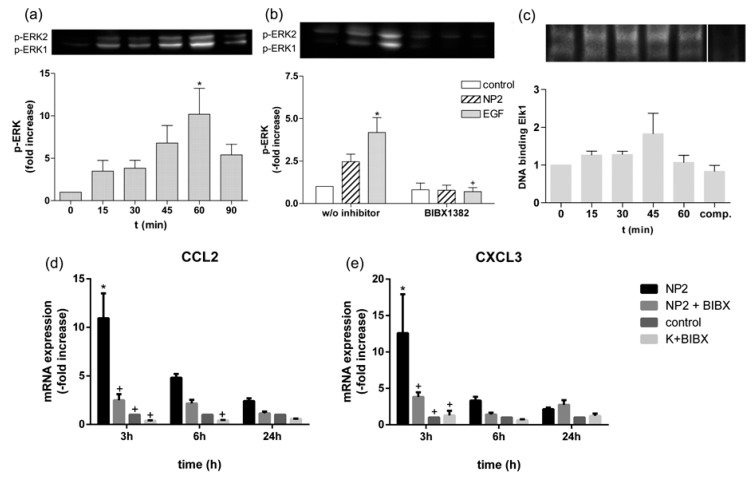
TiO_2_ NPs induced thee EGFR/ERK/ELK signaling pathway. (**a**) TiO_2_ NPs induced activation of p-ERK1/2. Caco-2 cells were stimulated with NP2 (concentration of 40 µg cm^−2^ of the cell growth surface) for 15, 30, 45, 60, and 90 min. Phosphorylated ERK1/2 was evaluated by Western blot analyses as shown in the upper row and presented in the bar diagram as the fold change to unstimulated samples = 0 min. Expression of p-ERK1/2 was normalized to total protein expression measured by densitometry; (**b**) the effect of EGFR inhibitor BIBX 1382 on NP- and EGF-induced ERK1/2 phosphorylation. Caco-2 cells were pre-treated with the inhibitor and exposed to NP2 or 100 ng mL^−1^ EGF for 30 min or remained untreated (w/o inhibitor). Treated samples are presented as the fold change to the untreated control sample; (**c**) TiO_2_ NPs induced binding capacity of ELK1. Caco-2 cells were stimulated with NP2 for 15, 30, 45, and 60 min. ELK1 binding was measured by EMSA and shown as the binding complex of ELK1 and ELK1-specific biotin-labeled oligonucleotides. For competition reaction (comp.) a 1000-fold molar excess of unlabeled oligonucleotides were used. Binding capacity was quantified by densitometry and treated samples are presented as the fold change to the untreated control. Presented are representative blots of *n* ≥ 3 independent experiments; (**d**,**e**) mRNA expression analyses of CCL2 (**d**) and CXCL3 (**e**) in Caco-2 cells. Caco-2 cells were pre-treated with 50 µM BIBX 1382 and exposed to NP2 or remained untreated for indicated time. The expression of CCL2 mRNA and the expression of CXCL3 mRNA was determined and values of the treated samples are presented as the fold change to the untreated control sample at the same time point. All data are presented the mean ± SEM of *n* ≥ 3 independent experiments. One- and two-way ANOVA, followed by Tukey’s multiple comparisons test (*p* < 0.05) were conducted. Asterisks (*) represent significant differences to untreated control w/o inhibitor, plus (+) represent significant differences to NP2 or EGF-treated cells without inhibitor.

To show that after endocytosis and transport via lysosomes gene expression is mediated via the known EGFR pathway, we analyzed the downstream elements of the pathway using Western blot and EMSA analyses. NP2 treatment of Caco-2 cells induced a transient activation of ERK1/2 compared to untreated control cells since levels of p-ERK1/2 increased in a time dependent manner until 60 min after NPs exposure. Activation of ERK1/2 faded 90 min after NPs exposure (Figure 7a). To analyze the involvement of EGFR in the NP-induced ERK activation we used EGFR inhibitor BIBX 1382. As shown by Western blot analysis p-EKR1/2 was induced 30 min after NPs and EGF stimulation by a factor of 2.5 ± 0.4 and 4.2 ± 0.9 compared to untreated control cells, and was completely abolished using BIBX 1382 (Figure 7b).

The best-studied nuclear target of phosphorylated ERK1/2 is the transcription factor ELK1 [20]. Stimulation with NP2 increased the binding of ELK1 to its DNA recognition site (Figure 7c) and declined 60 min after NPs treatment. Densitometric analyses revealed a 1.8 ± 0.5-fold increase of the binding complex compared to untreated control. Expression analysis revealed an increase by factor 11.0 ± 2.6 and 12.6 ± 5.3 3 h after NPs exposure of CCL2 and CXCL3 mRNA, respectively (Figure 7d). 6 h after NPs treatment mRNA expression of CCL2 and CXCL3 was induced by 4.8 ± 0.4-fold and by 3.3 ± 0.5-fold, respectively. Pre-incubation with EGFR inhibitor BIBX 1382 decreased the NP-induced mRNA expression of CCL2 and CXCL3 after 3 h by 77% and by 70%, and after 6 h by 54% and 58%.

## 3. Discussion

Although it has been shown that intestinal epithelial Caco-2 cells exposed to TiO_2_ NPs induced a transient inflammatory response, the interaction mechanism of TiO_2_ NPs with the cell surface remains unclear. By CLSM we were able to show that TiO_2_ NPs are internalized in Caco2-cells (Figure 3) and confirmed previously-published data using TEM [16,17,18,21]. The mechanism of internalization is still unknown. Some studies suggest that TiO_2_ NPs are internalized by endocytic mechanisms [12] or an interaction with the Toll-like receptor 4 [10,11]. To gain deeper insight into potential uptake mechanisms, we focused on endocytic pathways and EGFR, which is influenced by NPs [22,23].

As shown in Figure 1, four different mechanisms (micropinocytosis, clathrin-mediated endocytosis, caveolae-mediated endocytosis, and endocytosis independent of clathrin and caveolae [19]) are known in epithelial cells. Actin and microtubule dynamics are involved in all types of endocytosis [24,25]. Our results showed that TiO_2_ NP-induced NF-κB response was reduced by cytochalasin D, which inhibits actin polymerization [26,27] and in part by nocodazole, which depolymerizes microtubules [28,29] (Figure 4a). Expression analyses of typical inflammatory genes [30] confirm these observations: TiO_2_ NPs of 5 nm and 10 nm induced expression of different inflammatory markers, as shown in our previous study [9], and were reduced by treatment with cytochalasin D and nocodazole (Figure 4b). It can be concluded that an intact cytoskeleton is essential for internalization of TiO_2_ NPs in Caco2 cells as has been demonstrated in human keratinocytes [31], alveolar macrophages, [32] and fibroblasts [33].

Transport within cells requires forming of subcellular structures. In Caco-2 cells these vesicles can be formed caveolae-mediated [15], clathrin-mediated [34], dynamin-dependent [35], and clathrin- and caveolae-independent [36]. To investigate the mechanism involved, we used specific inhibitors of the key molecules: First, we used nystatin, a sterol-binding drug, which prevents the formation of lipid rafts [37]. Although there have been reports that nystatin inhibits cellular uptake of TiO_2_ NPs in Caco-2 cells [12] as well as in intestinal rainbow trout cells [38], we could not detect an altered cellular response to TiO_2_ NPs after treatment with nystatin (Figure 4) at concentrations which do not alter cell viability. Next, we treated Caco-2 cells with chlorpromazine. This cationic amphiphilic drug prevents coated pit assembly at the cell surface in clathrin-mediated endocytosis (CME) [39]. CME is characterized by the formation of a clathrin lattice around the invaginated membrane. This premature vesicle is pinched off by the GTPase dynamin to form clathrin-coated vesicles (CCV) [40]. We observed a reduction in the NP-induced NF-κB activation in Caco-2 cells (Figure 4a) but we could not detect an influence in the expression of IL8 mRNA and CXCL10 mRNA (Figure 4b). Therefore, we suggest the inflammatory response to TiO_2_ NPs in Caco-2 cells is only partly mediated by clathrin. This confirms a previous study, which has shown an involvement of CME in the uptake of TiO_2_ NPs in Caco-2 cells [12].

Clathrin-dependent endocytosis is mostly associated with receptor-mediated endocytosis [22], and NPs induced an EGFR mediated signaling pathway via extracellular signal-regulated kinase (ERK) in lung epithelial cells [41,42]. We, thus, examined the effect of EGFR kinase inhibitors BIBX1382 [23] and CL-387785 [43] to the cellular response of TiO_2_ NPs. Both block phosphorylation of tyrosine kinase and prevent activation of downstream signal transduction pathways. The NP-induced activation of NF-κB and mRNA expression of IL8, CCL20, and CXCL10 was decreased after treatment with EGFR inhibitors (Figure 5a,b). The results demonstrate that activation of EGFR is essential for initiating cellular response to TiO_2_ NPs. We assume TiO_2_ NPs activate EGFR and will be internalized into vesicles. As receptor-ligand interactions are very specific, it is most likely that recognition is not based on the metallic TiO_2_, but on some of the proteins present in the protein corona [44,45,46].

As activation of EGFR is required for cellular response to TiO_2_ NPs, we further analyzed downstream effectors of EGFR. ERK1/2 is part of the Ras/Raf/MEK/ERK signal transduction cascade [47]. Our results show that NPs induced phosphorylation of ERK1/2 (Figure 7). This activation was followed by an increased binding capacity of the transcription factor ELK1 and an increased mRNA expression of ERK1/2 target genes CCL2 and CXCL3 [48]. The NP-induced activation was reduced after EGFR inhibition. Various studies using different types of NPs have shown an activation of ERK signaling pathway [49,50] with a previous activation of EGFR in epithelial cells [51,52,53,54,55]. Further, it was shown that the expression of CCL2 and CXCL3 mRNA was increased after different inflammatory stimuli and was part of an inflammatory response, mediated by EGFR [56,57].

In conclusion, we show that in Caco-2 cells TiO_2_ NPs, probably due to their protein corona, are recognized by EGFR, which is internalized via clathrin-dependent and clathrin-independent mechanisms, but not by caveolin-mediated endocytosis. The involvement of cytoskeletal, as well as dynamin, in this endocytosis process has been documented. TiO_2_ NPs activate the complete EGFR/ERK/ELK signaling pathway, including the expression of the effector mRNA CCL2 and CXCL3.

## 4. Material and Methods

### 4.1. Preparation of TiO_2_ NPs

Titanium (IV) dioxide (anatase) particles were obtained from Alfa Aesar (Alfa Aesar GmbH and CoKG, Karlsruhe, Germany). Four differently-sized particles were used: 1. 5 nm (NP1; Stock Number 44689, Lot F11T023, specific surface area (SSA) 210 m^2^ g^−1^), 2. 10 nm (NP2; Stock Number 44690, Lot B19T020; SSA 120 m^2^ g^−1^), 3. 32 nm (NP3; Stock Number 39953, Lot F23T043, SSA 51 m^2^ g^−1^) and 4. 490 nm (NP4; Stock Number 36199, Lot G02S013; SSA not specified). Stock dispersions of all particles were prepared with deionized water to a final concentration of 2 mg mL^−1^. The dispersion was sonicated at 23 KHZ and 150 W (MSE Ltd., London, UK) for 2 min and finally autoclaved. Immediately before treatment of the cells the dispersion was diluted in culture medium as indicated below.

### 4.2. Nanoparticle Characterization

Characterizations of the particles were performed by transmission-electron microscopy (TEM) using freeze-fracture preparation technique and by static light scattering (SLS). Sonicated and autoclaved stock solutions of NPs were diluted 1:10 in cell culture medium and cryoprotected by immersion in glycerol solution. Samples were cryofixed into melting Freon 22 and liquid nitrogen. Freeze fracturing took place at −120 °C with a BAF 400 (Bal-Tec, Balzers, Liechtenstein). Freeze-fractured specimens were replicated by application of Pt/C and C by electron-gun evaporation. The replicas were cleaned in concentrated sodium hypochlorite and in acetone and examined with a Tecnai 10 (FEI Company, Hillsboro, OR, USA) transmission-electron microscope operated at 80 kV. Size measurement of single particles and particle aggregations using the TEM images was conducted with the program “Bild-Vermessen 1.0” (CAD-KAS Kassler Computersoftware GbR, Markranstädt, Germany). At least 30 single particles and 20 aggregates of the different NPs were measured. Particle size distribution was measured by SLS using a LS 230 (Beckmann Coulter, Krefeld, Germany). The particle dispersion was dosed into the instrument without special sample preparation. The volume fraction-length mean diameter was measured.

### 4.3. Cell Culture

Human colon adenocarcinoma cell line Caco-2 (Toni Lindl, Munich, Germany) between passage 24 and 50 were cultured in Caco-2 medium (45% Dulbecco’s Modified Eagle Medium (DMEM), low glucose, 45% Ham’s F12, 9% fetal calf serum (FCS), 0.9% non-essential amino acids) (all PAA Laboratories GmbH, Austria), and insulin (10 µg mL^−1^) (Biochrome AG, Berlin, Germany) at 37 °C and 5% CO_2_. For all experiments cells were seeded at a density of 1× 10^5^–2 × 10^5^ cells cm^−2^. TiO_2_ particles were added to the cells at a final concentration of 40 µg cm^−2^ cell growth surface as this concentration is in accordance with the observed exposure in humans [2] and in the range of other comparable references [3,5,18,58]. We determined the time we exposed the cells to NPs prior to the described experiments by a number time course analyses.

### 4.4. mRNA Expression Analysis

For mRNA expression analysis cells were seeded in 24-well or six-well culture plates (Sarstedt AG and Co., Nümbrecht, Germany). After reaching confluency, TiO_2_ particles of indicated sizes or water (control) were added at a final concentration of 40 µg cm^−2^ cell growth surface. At indicated time points cells were washed twice with PBS and proceeded to RNA extraction. RNA was extracted using the GeneJET RNA Purification Kit (Thermo Fisher Scientific GmbH, Dreieich, Germany) and first-strand cDNA synthesis was prepared using a RevertAid™ H Minus First Strand cDNA Synthesis Kit (Thermo Fisher Scientific GmbH, Dreieich, Germany) as described by the manufacturer. Real-time PCR was performed on a 7500 Real-Time PCR Systems (Life Technologies Inc., Carlsbad, CA, USA) using HOT FIREPol^®^ EvaGreen^®^ qPCR Mix Plus ROX (Solis BioDyne, Tartu, Estonia). Sequences of primers (used at 0.2 µM) were as follows: B2M (Beta-2 microglobulin) forward: GCAAGGACTGGTCTTTCTATCT, reverse: TAACTATCTTGGGCTGTG-ACAAA; CCL2 (chemokine (C–C motif) ligand 2) forward: CCCAAAGAAGCTGTGATCTTCA; reverse: TCTGGGGAAAGCTAGGGGAA, CCL20 (chemokine (C–C motif) ligand 20) forward: CGAATCAGAAGCAGCAAGCAA, reverse: TTGCGCACACAGACAACTTT; CXCL3 (chemokine (C–X–C motif) ligand 3) forward: CCCAAACCGAAGTCATAGCCA, reverse: ACCCTGCAGGAAG-TGTCAA; CXCL10 (chemokine (C–X–C motif) ligand 10) forward: GCCATTCTGATTTGCTGCCTT, reverse: GCTCCCCTCTGGTTTTAAGGA; GAPDH (glyceraldehyde 3-phosphate dehydrogenase) forward: AGAGCACAAGAGGAAGAGAGAG, reverse: GGTTGAGCACAGGGTACTTTATT; IL8 (interleukin 8) forward: CACCGGAAGGAACCATCTCA, reverse: TGGCAAAACTGCACCTTC-ACA. Parameters for qPCR were as follows: 95 °C for 15 min, 40 cycles of 10 s at 95 °C, 30 s at 60 °C, and 30 s at 72 °C. After cycling, melting curve analysis was performed. Expressions of the different genes was normalized to the expressions of GAPDH and B2M and compared to control.

### 4.5. NF-κB Reporter Gene Assay

Caco-2^nfkb-RE^ [9] cells were seeded in 96-well plates (Corning Incorporated, New York, NY, USA). At confluency, inhibitors of endocytosis were added to the cells and incubated as indicated below. Subsequently, cells were treated with TiO_2_ NPs or water (control) at a final concentration of 40 µg cm^−2^ cell growth surface. After 6 h cells were harvested and luciferase activity was measured for 5 s in a CHAMELEON™ V plate reader (Hidex, Finland) using Beetle-Juice substrate (PJK GmbH, Kleinblittersdorf, Germany) according to the manufacturer’s instructions. Luciferase activity is expressed in relation to untreated controls. 

### 4.6. Fluorescence and Confocal Laser Scanning Microscopy (CLSM)

Detection of lysosomes was performed by fluorescence microscopy. Caco-2 cells were seeded in eight-well on cover glass II (Sarstedt AG and Co., Nümbrecht, Germany). At confluency, prepared medium with TiO_2_ NPs or water (control) was added. Simultaneously LysoTracker^®^ Red DND-99 (Molecular Probes, Eugene, OR, USA) was added to medium at a final concentration of 50 nM. After incubation time of 3 h cells were observed by fluorescence microscopy. Co-localization of lysosomes and NPs was detected by different filter sets. NPs were visualized by using FITC filter set and LysoTracker^®^ Red DND-99 by using filter set appropriate to Texas Red^®^ dye. To investigate intracellular distribution of the NPs, confocal laser scanning microscopy was employed. Caco-2 cells were seeded in a removable 12-well chamber (ibidi GmbH, Munich, Germany). At confluency, prepared medium with TiO_2_ NPs or water (control) was added. After incubation time of 6 h cells were harvested and stained for actin with Alexa Fluor 633 Phalloidin (Molecular Probes, Eugene, OR, USA) as described by the manufacturer. Nuclei were stained with Dapi-Fluoromount-G™ clear mounting media (SouthernBiotech™, Birmingham, AL, USA). NPs were excited by a laser at 488 nm and the emission was measured from 490 nm to 501 nm. Excitation of phalloidin took place by a laser at 633 nm and emission was measured from 642 nm to 655 nm to allow a clear separation of both signals. Confocal and fluorescence images were acquired with a confocal laser scanning microscope TCS SP8 (Leica Microsystems GmbH, Wetzlar, Germany) using a 40× objective and 63× glycerol immersion objective.

### 4.7. Western Blot Analyses

Caco-2 cells were seeded in six-well plates (8.87 cm^−2^ per well) (Sarstedt, Germany) at a density of 1–2 × 10^5^ cells cm^−2^. At confluency, prepared medium with 10 nm TiO2 NPs was added to the cells at a final concentration of 40 µg cm^−2^ cell growth surface or remained untreated. EGF at a concentration of 100 ng mL^−1^ was used as a positive control in the EGFR/ERK signaling pathway as described [59]. Cells were washed twice with cold PBS and lysed 20 min on ice in RIPA buffer (Sigma-Aldrich, St. Louis, MO, USA) added with inhibitors (1 mM PMSF, 1 mM Na_3_VO_4_, 1× complete protease inhibitor cocktail (Santa Cruz Biotechnology, Santa Cruz, CA, USA), 1× phosphatase inhibitor cocktail 3 (Sigma-Aldrich, St. Louis, MO, USA). Lysates were clarified by centrifugation (12,000× *g* for 6 min) and protein concentration was measured using bicinchoninic acid assay (BCA) reagent. 40 µg protein from each preparation were separated by sodium dodecyl sulfate-polyacrylamide gel electrophoresis (SDS-PAGE) and transferred to a nitrocellulose membrane (Carl Roth GmbH + Co.KG, Karlsruhe, Germany). Membranes were blocked in 5% (*w*/*v*) BSA/TBST (20 mM Tris–HCl (pH 7.6), 150 mM NaCl, and 0.1% (*v*/*v*) Tween 20) for 1 h at room temperature. Primary antibody incubation p-ERK1/2 1:200 (sc-7383, Santa Cruz Biotechnology, Santa Cruz, CA, USA) in 5% BSA/TBST took place at 4 °C overnight, followed by secondary antibody incubation 1:500 in TBST (anti-mouse HRP (PAB10782, Abnova, Taiwan)) for 2 h at room temperature. The antibody was detected with enhanced chemiluminescence (ECL) reagent (MBL International Corporation, Woburn, MA, USA) and visualized with Fusion Solo S (Vilber Lourmat Deutschland GmbH, Eberhardzell, Germany). Densitometry of the bands was analyzed using FusionCapt Advance Solo 4s software and the ratio of p-ERK to total protein, detected by Ponceau staining, was determined. Expressions of the treated samples were set in relation to the untreated control sample. Experiments were performed at least three times.

### 4.8. Electrophoretic Mobility Shift Assay (EMSA)

Binding of ELK1 was investigated by non-radioactive EMSA using a LightShift EMSA Optimization and Control Kit (Thermo Fisher Scientific Inc., Waltham, MA, USA) according to manufacturer’s protocol. In detail, Caco-2 cells were seeded in six-well plates (Sarstedt AG and Co, Nümbrecht, Germany) at a density of 1–2 × 10^5^ cells cm^−2^. At confluency, cells were stimulated with 10 nm TiO_2_ NPs at a final concentration of 40 µg cm^−2^ cell growth surface or remained unstimulated. Cells were washed twice with ice cold PBS and lysed 20 min on ice in lysis buffer (10 mM HEPES, 1.5 mM MgCl_2_, 10 mM KCl, 1 mM DTT, 1 mM PMSF, 1× complete protease inhibitor cocktail (Santa Cruz Biotechnology, Santa Cruz, CA, USA)) adapted from [60]. Cells were scraped, placed in a microliter tube, and centrifuged 20 min at 11,000× *g* at 4 °C to separate cytosolic and nuclear fractions. Supernatant was decanted and pellet was suspended in extraction buffer to lyse nuclei (20 mM HEPES, 1.5 mM MgCl_2_, 0.42 M NaCl, 0.2 mM EDTA, 25% (*v*/*v*) glycerol, 1 mM DTT, 1 mM PMSF, 1× complete protease inhibitor cocktail, [60]). 2 µg of nucleus extraction were used for binding reaction with biotin-labeled DNA for ELK1: 5′-TTTGCAAAATGCAGGAATTGTTTTCACAGT-3′ [61]. Samples were separated by 6% native polyacrylamide gel and transferred to a nylon membrane (GE Healthcare, Little Chalfont, UK). Biotin was detected with peroxidase-coupled streptavidin and ECL (Thermo Fisher Scientific Inc., Waltham, MA, USA) and visualized and quantified with Fusion Solo S (Vilber Lourmat Deutschland GmbH, Eberhardzell, Germany). Densitometry of the binding complex was analyzed using FusionCapt Advance Solo 4s software and expressions of the treated samples were set in relation to the untreated control sample. Experiments were performed at least three times.

### 4.9. Cell Treatment

To investigate the mechanisms involved in the TiO_2_ NPs-induced inflammatory response in Caco-2 cells, we used various inhibitors of endocytic processes (Figure 1, Table 1). Confluent monolayers of Caco-2 cells were pre-incubated with inhibitors (cytochalasin D (AppliChem, Darmstadt, Germany), nocodazole (AppliChem, Darmstadt, Germany), nystatin (AppliChem, Darmstadt, Germany), dynasore (Santa Cruz Biotechnology, Inc., Santa Cruz, CA, USA), chlorpromazine (Sigma-Aldrich, St. Louis, MS, USA), BIBX 1382 (Santa Cruz Biotechnology, Inc., Santa Cruz, CA, USA), and CL-387785 (Santa Cruz Biotechnology, Inc., Santa Cruz, CA, USA)). The final concentrations that were used in medium, incubation time, and effect of inhibitors are summarized in Table 1. After pre-incubation, the medium with TiO_2_ NPs or water (control) was added at a final concentration of 40 µg cm^−2^ cell growth surface and incubated for 3 h or 6 h. Cytotoxic activity was tested by the ToxiLight™ bioassay kit (Lonza Group AG, Walkersville, MD, USA). No cytotoxic effect was observed for inhibitors at the used concentration (data not shown). After particle treatment, cells were washed twice with PBS and further treated with the stated methods. 

Epithelial growth factor (EGF) at a concentration of 100 ng mL^−1^ was used as a positive control in the EGFR/ERK signaling pathway, as described [61]. A confluent monolayer of Caco-2 cells was incubated for 1 h with 50 µM BIBX 1382 and subsequently stimulated with NPs, EGF, or left unstimulated. After treatment, cells were washed twice with cold PBS and processed as described above. Results of the treated cells are presented as the fold change to untreated cells.

### 4.10. Data Analysis

We performed every experiment with, at least, replicate samples for each treatment with the appropriate controls carried out on three different days (i.e., at least technical duplicates with three biological replicates). Data of RT-qPCR were analyzed using the delta-delta *C_t_* method [65]. Results are presented as means ± standard error (SEM) and expressed as the fold change to the untreated control. Statistical analysis was carried out using GraphPad Prism 4 software (GraphPad Software, Inc., La Jolla, CA, USA). After testing for Gausian distribution (Kolmogorov-Smirnoff test) and homogeneity of variance (Levene test) comparisons of the groups were either carried out by non-parametric analysis, followed by Bonferroni post-tests or by ANOVA, followed by Tukey’s multiple comparison test. Differences were considered to be statistically significant at *p* < 0.05. 

## Figures and Tables

**Table 1:** Inhibitors used in this study. Presented are the effects of the inhibitors, the corresponding reference for the used concentration, and pre-incubation time.

Inhibitor	Effect	Reference	Concentration	Preincubated Time
Cytochalasin D	Inhibits actin polymerization	[26,27]	0.1 µg mL^−1^	30 min
Nocodazole	Depolymerizes microtubules	[28,29]	2 µM	30 min
Nystatin	Inhibits Caveolae-dependent endocytosis	[37]	50 µg mL^−1^	30 min
Dynasore	Inhibits dynamin	[62,63]	100 µM	1 h
Chlorpromazine	Inhibitor of clathrin-mediated endocytosis	[35,64]	100 µM	1 h
BIBX 1382	EGFR kinase inhibitor	[23]	46 µM	1 h
CL-387785	EGFR kinase inhibitor	[43]	20 µM	3 h
